# Application of Defined Approaches for Skin Sensitization to Agrochemical Products

**DOI:** 10.3389/ftox.2022.852856

**Published:** 2022-05-02

**Authors:** Judy Strickland, James Truax, Marco Corvaro, Raja Settivari, Joseph Henriquez, Jeremy McFadden, Travis Gulledge, Victor Johnson, Sean Gehen, Dori Germolec, David G. Allen, Nicole Kleinstreuer

**Affiliations:** ^1^ Integrated Laboratory Systems, LLC, Research Triangle Park, NC, United States; ^2^ Corteva Agriscience, Regulatory Sciences R&D, Rome, Italy; ^3^ Corteva Agriscience, General, Genetic, and Molecular Toxicology, Newark, DE, United States; ^4^ Corteva Agriscience, Regulatory Toxicology and Risk Group, Indianapolis, IN, United States; ^5^ Burleson Research Technologies, Inc., Morrisville, NC, United States; ^6^ Systems Toxicology Branch, Division of the National Toxicology Program, National Institute of Environmental Health Sciences, Research Triangle Park, NC, United States; ^7^ National Toxicology Program Interagency Center for the Evaluation of Alternative Toxicological Methods, Division of the National Toxicology Program, National Institute of Environmental Health Sciences, Research Triangle Park, NC, United States

**Keywords:** adverse outcome pathway, alternatives to animal testing, chemical allergy, defined approaches, new approach methodologies, skin sensitization, agrochemicals

## Abstract

Skin sensitization testing is a regulatory requirement for safety evaluations of pesticides in multiple countries. Globally harmonized test guidelines that include *in chemico* and *in vitro* methods reduce animal use, but no single assay is recommended as a complete replacement for animal tests. Defined approaches (DAs) that integrate data from multiple non-animal methods are accepted; however, the methods that comprise them have been evaluated using monoconstituent substances rather than mixtures or formulations. To address this data gap, we tested 27 agrochemical formulations in the direct peptide reactivity assay (DPRA), the KeratinoSens™ assay, and the human cell line activation test (h-CLAT). These data were used as inputs to evaluate three DAs for hazard classification of skin sensitization potential and two DAs for potency categorization. When compared to historical animal results, balanced accuracy for the DAs for predicting *in vivo* skin sensitization hazard (i.e., sensitizer vs. nonsensitizer) ranged from 56 to 78%. The best performing DA was the “2 out of 3 (2o3)” DA, in which the hazard classification was based on two concordant results from the DPRA, KeratinoSens, or h-CLAT. The KE 3/1 sequential testing strategy (STS), which uses h-CLAT and DPRA results, and the integrated testing strategy (ITSv2), which uses h-CLAT, DPRA, and an *in silico* hazard prediction from OECD QSAR Toolbox, had balanced accuracies of 56–57% for hazard classification. Of the individual test methods, KeratinoSens had the best performance for predicting *in vivo* hazard outcomes. Its balanced accuracy of 81% was similar to that of the 2o3 DA (78%). For predicting potency categories defined by the United Nations Globally Harmonized System of Classification and Labelling of Chemicals (GHS), the correct classification rate of the STS was 52% and that of the ITSv2 was 43%. These results demonstrate that non-animal test methods have utility for evaluating the skin sensitization potential of agrochemical formulations as compared to animal reference data. While additional data generation is needed, testing strategies such as DAs anchored to human biology and mechanistic information provide a promising approach for agrochemical formulation testing.

## 1 Introduction

Safety assessment of agrochemicals, either as single active ingredients or end-use product formulations, which are typically multiconstituent substances with defined compositions, requires extensive testing for hazard characterization and risk assessment purposes. In the agrochemical sector, acute toxicity testing is usually required for hazard classification used to develop appropriate labeling for safe transport, handling, and use (Corvaro et al., 2017). Studies performed for the safety assessment of agrochemical formulations are referred to colloquially as the acute toxicity “6-pack,” which includes tests for acute systemic toxicity by the oral, dermal, and inhalation routes; and acute topical toxicities of eye and skin irritation and skin sensitization. Data from these tests are required according to international guidance on chemical safety such as those issued by the United Nations (UN, 2021a; UN, 2021b), which are implemented regionally. Investigating skin sensitization potential is one of the mandatory global requirements for agrochemical formulations that influences labeling and, more recently, quantitative risk assessment (UK, 2008; Sanvido et al., 2018).

Traditionally, guinea pig methods, such as the Buehler or the adjuvant-based maximization test, were used for evaluating sensitization potential and both are included in Organisation for Economic Co-operation and Development (OECD) test guideline (TG) 406 (OECD, 1992). However, the methods described in TG 406 were never formally validated and provide limited information on potency (Van Loveren et al., 2008). They also incorporate experimental procedures that have ethical implications for animal welfare, including a sensitization phase that may use an adjuvant and final elicitation of an adverse allergy event. These limitations, as well as concern for the sensitivity of the assays, have resulted in their reduced acceptance in some jurisdictions specifically for some regulatory contexts within the European Union (e.g., EU for REACH), although they are still preferred in others (e.g., Asian and South Pacific countries) (Daniel et al., 2018). An alternative to the guinea pig methods, the murine local lymph node assay (LLNA) (OECD, 2010a), is the most commonly used *in vivo* method for skin sensitization testing required for agrochemical formulations. The LLNA uses fewer animals and has a shorter exposure duration than the guinea pig methods. Because it measures an early event of the sensitization phase, it reduces the pain and distress that would be produced by an adverse skin reaction. Since the assay measures lymph node T-cell proliferation, it also provides a quantitative assessment of potency. In the United States, the LLNA was formally recommended by the Interagency Coordinating Committee on the Validation of Alternative Methods as a reduction and refinement alternative to the guinea pig test methods (NIH, 1999). The LLNA was internationally accepted via OECD TG 429 (OECD, 2010a), and non-radiolabel modifications were later adopted in TG 442A and TG 442B (OECD, 2010b; OECD, 2014).

Despite the reduction and refinement advantages of the LLNA, it has a number of known limitations, which include false negatives for some metals and clinically relevant allergens and false positives for known irritants (NIH, 2011; Roberts et al., 2016a; Roberts et al., 2016b; Roberts et al., 2016c; Roberts and Api, 2018). These limitations, combined with the greater number of animals required for skin sensitization testing relative to other 6-pack endpoints, have motivated the development and use of non-animal alternatives. Furthermore, advances in knowledge of the mechanisms for skin sensitization makes the use of established alternative methods to address the skin sensitization endpoint an achievable milestone in the overall goal to reduce and ultimately replace animal use in the agrochemical space.

The adverse outcome pathway (AOP) for skin sensitization initiated by covalent binding of chemicals, including agrochemicals, to skin proteins is well-characterized and has been described in an OECD monograph (OECD, 2012). The AOP includes four key events that must proceed for a skin sensitization response to develop. Key event 1 (KE1) is the formation of protein adducts via covalent bonding of a chemical to amino acids in skin cells. KE1 is addressed by three *in chemico* assays described in TG 442C (OECD, 2021c). These assays measure the reactivity of chemicals with synthetic peptides containing lysine and cysteine, which can be used to discriminate between sensitizers and nonsensitizers. Key event 2 (KE2) is the induction of an inflammatory response in keratinocytes, the most common cells in skin. The KeratinoSens and LuSens assays described in TG 442D (OECD, 2018a) address KE2 by measuring the induction of a transfected luciferase gene under the control of the antioxidant response element in a keratinocyte cell line. KE3, dendritic cell activation, is addressed by the human cell line activation test (h-CLAT) and U937 cell line activation test, U-SENS™, covered in TG 442E (OECD, 2018b). These tests measure changes in the levels of T-cell surface costimulatory proteins CD86 and CD54 as an indicator of dendritic cell activation. Key event 4 (KE4) is the proliferation of T-cells, which depends on activation of keratinocytes (KE2) and/or dendritic cells (KE3) and is measured by the LLNA. Currently, there are no accepted non-animal alternatives to address KE4.

While internationally accepted, the TGs that measure the first three KEs are not designed to be “stand-alone” replacements for *in vivo* assays because the individual outcomes are not necessarily predictive of the overall adverse outcome of skin sensitization. However, this limitation can potentially be overcome by assessing the totality of available experimental and expert-based evidence within the context of integrated approaches to testing and assessment (IATA). However, use of IATA may require expert judgment to reach a conclusion on skin sensitization potential. In addition, the conclusion reached using IATA may not be considered harmonized under the OECD Mutual Acceptance of Data agreement, whereby data developed for a regulatory program in one country is also acceptable in other OECD member countries (OECD, 1981). These limitations may be overcome by the application of defined approaches (DAs). A DA consists of a fixed data integration procedure applied to a defined combination of test results with no expert judgment needed to interpret the outcome (OECD, 2017). Increased acceptance of DAs has resulted in their recent implementation in an OECD guideline, Guideline 497 (OECD, 2021b), that is covered by the Mutual Acceptance of Data agreement. This guideline incorporates three specific DAs. The “2 out of 3” (2o3) DA relies on two concordant tests for hazard classification from the direct peptide reactivity assay (DPRA), KeratinoSens, and h-CLAT. The guideline also describes two versions of an integrated testing strategy (ITS) that uses h-CLAT, DPRA, and an *in silico* hazard prediction. ITSv1 uses Derek Nexus (Lhasa Limited) for the *in silico* prediction and ITSv2 uses OECD’s QSAR Toolbox. Both ITSv1 and ITSv2 include a scoring system that can be used to classify substances into the potency categories of the United Nations Globally Harmonized System of Classification and Labelling of Chemicals (GHS) (UN, 2021a).

With the introduction of OECD Guideline 497 on DAs for skin sensitization testing (OECD, 2021b), there is a need for international harmonization in the testing of agrochemical formulations using individual *in vitro* test methods and DAs. For example, in 2018, the United States Environmental Protection Agency started accepting predictions from two DAs for hazard classification of monoconstituent substances: the 2o3 and the KE 3/1 sequential testing strategy (STS) (US EPA, 2018). However, most pesticides are sold as formulated products rather than single compounds. Coformulant compounds range in their purpose, but can include active ingredients, emulsifiers, wetting agents, stabilizers, antimicrobials, safeners, adjuvants, solvents, diluents, binders, fertilizers, and clays. A recent survey described more than 60 agrochemical formulation types, including mixtures of multiple formulation types (Croplife International, 2017). The net result is that most agricultural products come in a variety of complex compositions, which can be roughly divided into liquid solvent-based, liquid water-based, and solid formulations. The *in vitro* tests available to address KE1, KE2, and KE3 have not been validated with complex mixtures, and verification of the applicability domain for agrochemical mixtures has been limited (Settivari et al., 2015). Currently, OECD Guideline 497 is restricted in applicability to monoconstituent substances and excludes formulation products due to lack of data characterizing the reliability of applying DAs to such mixtures (OECD, 2021b).

The purpose of this research was to investigate a potential extension of the applicability domain of three accepted non-animal OECD test methods, the DPRA (TG 442C), the KeratinoSens (TG 442D) and the h-CLAT (TG 442E) and three DAs, the 2o3, STS, and ITSv2, to complex mixtures by testing a balanced selection of agrochemical formulations with known skin sensitization potential. Demonstration of the applicability of these testing approaches to such materials would be expected to support their greater use for agrochemical product registration and result in a decrease in the use of animals for this purpose.

## 2 Materials and Methods

### 2.1 Formulations Tested

A total of 27 agrochemical formulations were provided to the National Toxicology Program by Corteva Agriscience for evaluation of sensitization potential using *in vitro* approaches. *In vitro* testing was performed by Corteva Agriscience (DPRA and KeratinoSens) and Burleson Research Technologies, Inc. (h-CLAT). These commercial formulations were sourced from several manufacturing sites globally. All formulations were liquids and were selected to cover the most commonly used formulation types. Specifically, 13 formulations were water-based liquids (six suspension concentrates and seven soluble liquids) and 14 were solvent-based (nine emulsion concentrates, three oil dispersions, and three emulsions in water). The complete compositions of these formulations are proprietary and cannot be disclosed. However, identity and percentage weight of each active substance and other selected composition information is reported in Supplementary File 1. For the purposes of this publication, the formulations are coded.

As described in the following section, formulations were also selected to provide a balanced representation of nonsensitizers and sensitizers.

### 2.2 *In Vivo* Reference Data

No new animal tests were conducted to obtain *in vivo* reference data. Instead, data were compiled from previously conducted *in vivo* animal skin sensitization studies used for registration purposes. These data, provided in Supplementary File 2, served as the reference data for classification of individual test materials according to GHS skin sensitization potency categories (UN, 2021a). All *in vivo* assays were considered valid; the positive control substances tested in these assays were within expected ranges.

LLNA studies compliant with OECD TG 429 and conducted between 2005 and 2012 were available for 18 formulations (7 positive, 11 negative). CBA/J mice were used for 15/18 formulations and BALB/c mice were used for 3/18 formulations. Formulations were applied to the ears of the mice in 1% L92 solution in water, except for one formulation, Dow1, for which propylene glycol was used as the vehicle. The vehicle of 1% L92 is recommended by the TG and is preferred for agrochemical formulations because it is water-based and simulates the common use condition of dilution with water for distribution via spray tanks. All studies were preceded by an irritancy screening for appropriate selection of the maximum dose. The dose spacing was not always compliant with the recommended dose spacing in TG 429, but this deviation from the guideline did not impact determination of potency. EC3 values, the effective concentration that produced a stimulation index of three, the threshold for a positive response, were interpolated using response data above and below the stimulation index of three. There was no need to rely on an extrapolation procedure which makes assumptions about the slope of the dose-response curve beyond the measured data.

Data from guinea pig maximization tests conducted between 1998 and 2009 in compliance with OECD TG 406 were available for five formulations (two positive, three negative) (OECD, 2021a). Each test included preliminary irritancy screens via both intradermal injection and epidermal application routes. The intradermal induction included Freund’s Complete Adjuvant and the epidermal induction used sodium lauryl sulfate.

Data from Buehler tests conducted between 1989 and 2000 in compliance with OECD TG 406 were available for four formulations (three positive, one negative) (OECD, 2021a). Tests included a preliminary irritancy screen and a three-induction experimental scheme.

When multiple tests where available for the same formulation, preference was given to LLNA data over guinea pig data and to guinea pig maximization test data over Buehler test data. LLNA is preferred because it provides a quantitative response measurement and a dose-response assessment. Two formulations had a negative result in the LLNA, with a concordant negative Buehler (Dow2) or guinea pig maximization test (Dow25). One formulation with a positive LLNA but a discordant negative Buehler test was assigned an overall classification of positive (Dow9).

Of the 27 formulations, 15 were GHS Not Classified (nonsensitizers), 11 were GHS Category 1B (other than strong) sensitizers and one was a GHS Category 1A (strong) sensitizer. Note that severe sensitization is a rare outcome in agrochemical formulations (Corvaro et al., 2017).

Finally, no human experimental data (i.e., skin sensitization patch tests) were available for any of the formulations tested. Adverse reporting data were reviewed; however, due to the sparsity of data, no evidence of skin sensitization reactions by any of the formulations in this paper could be inferred. Hence, reference classification was solely based on evidence from existing animal assays.

### 2.3 Individual Non-Animal Methods for Skin Sensitization

For the *in vitro* assays, the formulations tested were considered to have a purity of 100% and a density of 1 g/ml.

#### 2.3.1 Direct Peptide Reactivity Assay (DPRA)

DPRA predicts the molecular initiating event, KE1, in the AOP for dermal sensitization. The assay was performed at Corteva Agriscience facilities according to TG 442C (OECD, 2021c) with few modifications. While TG 442C considers DPRA to be technically applicable for testing multiconstituent substances and mixtures, testing at the specified molar concentration (100 mM) is not possible. Therefore, we used a modified approach wherein the doses were based on considering each formulation as a single entity rather than as a mixture of multiple components. In this approach, a common molecular weight (MW) of 400 Da was assumed for each formulation, consistent with the approximate MW of the agrochemical active ingredients tested in the present study. The same approach was followed in a recent study testing agrochemical formulations in an *in vitro* KeratinoSens assay (Settivari et al., 2015). The use of a *pro forma* molecular weight for substances with no defined molecular weight was originally proposed in the KeratinoSens protocol (ECVAM, 2019) based on an average molecular weight of 200 Da for cosmetic ingredients.

The positive control used to assess run acceptance in these studies was 100 mM cinnamic aldehyde in acetonitrile. The purity of the synthetic peptides used in the assay, acetylated lysine (Ac-RFAKAA-COOH) or acetylated cysteine (Ac-RFAACAA-COOH) (Celtek Bioscience, Franklin, TN), was 98% or higher.

To conduct the assay, formulations were combined with the cysteine- and lysine-containing peptides at ratios of 1:10 and 1:50, respectively. Three replicates of these solutions were incubated for 24 h in the dark at 25 ± 2.5°C. The concentrations of the cysteine- and lysine-containing peptides were then measured using high performance liquid chromatography with gradient elution and UV detection at 220 nm. The average percent depletion of the cysteine- or lysine-containing peptides replicates was calculated by comparing the concentrations of solutions with and without the respective test materials. Then cysteine and lysine depletion values were averaged together. To confirm potential quantitative interference with the test compound in UV monitoring, we also assayed a preparation containing only test substance without cysteine or lysine peptide stock. Based on the OECD TG 442C criteria, we classified a test substance as positive if the average lysine/cysteine depletion was 6.38% or higher.

OECD TG 442C only describes UV determination of peptide depletion. To improve assay specificity, we also measured peptide depletion using in-line selected ion monitoring for mass spectra of both cysteine and lysine peptides after UV detection. This added step facilitated accurate peptide depletion measurements in the event of co-elution of test chemical and peptide as well as monitoring of cysteine and lysine peptide dimer formation. Intact peptide mass-to-charge ratios were monitored for both the cysteine and lysine peptides. Quantitation was performed on the [M+2H]2+ (376 Da) for the cysteine peptide, while the [M + H]+ (776 Da) was used for the lysine peptide. The retention time was observed to be approximately 12.8 min for the cysteine and 10.2 min for the lysine peptide standards. We report mass spectra results when UV results indicated test chemical interference with the peptide peak determination.

#### 2.3.2 KeratinoSens Test Method

KeratinoSens is an *in vitro* skin sensitization assay addressing the AOP KE2 relevant to keratinocyte responses, including activation of inflammatory cytokines and induction of cytoprotective genes. The KeratinoSens cell line was kindly provided by Dr. Andreas Natsch (Givaudan Schweiz AG, Switzerland). The KeratinoSens test method was performed at Corteva Agriscience facilities as described by Settivari et al. (2015) and adopted by OECD in TG 442D (OECD, 2018a) with minor modifications. Consistent with our approach for the DPRA, a *pro forma* MW of 400 Da was assumed for each formulation. Each test substance was tested at 12 concentrations ranging from 0.4 to 800 μg/ml (instead of 0.2–400 μg/ml suggested in TG 442D). To enable comparison of KeratinoSens data for each formulation in relation to its corresponding active ingredient, the data are presented in μM units (i.e., 1–2,000 μM). The positive control used in these studies was a two-fold dilution in dimethyl sulfoxide (DMSO) of cinnamic aldehyde tested at five concentrations ranging from 4 to 64 µM. The negative control was complete Dulbecco’s modified Eagle’s medium supplemented with 9.1% fetal bovine serum and 0.55 mg/ml Geneticin^®^ (GIBCO) with 1% DMSO. A no-cell blank was also included. Luminescence was measured with a FLUOstar^®^ Omega (BMG LABTECH, Inc.) multidetection microplate reader to assess luciferase activity. The average maximum fold induction of luciferase activity observed at any concentration of the test substance and the positive control were determined, as well as the concentration of test material that increased luciferase activity to 1.5-fold (EC1.5). In addition, cell viability was determined using the MTT assay in which reduction of the yellow tetrazolium dye [3-(4,5-dimethylthizol-2-yl)-2,5-diphenyltetrazolium bromide) to a purple formazan product was assessed by measuring absorbance with a spectrophotometer. All agrochemical formulations were tested in two or more wells for each replicate and each experiment was repeated on at least two separate days (independent replicates). Each replicate was considered acceptable when all of the following criteria were met: 1) the positive control induced a dose-dependent increase in luciferase activity with EC_1.5_ between 7 and 30 μM; 2) maximum luciferase induction at 64 µM was between 2- and 8-fold; and 3) average coefficient of variation of the luminescence reading for the solvent-control wells was less than 20%. A test substance was considered positive for skin sensitization when all of the following conditions were met:

• Average maximum fold induction of luciferase activity was at least 1.5-fold over the solvent control value.
• Cell viability was greater than 70% at the lowest concentration with induction of luciferase activity at greater than or equal to 1.5-fold.
• The EC1.5 value was less than 1,000 µM.
• There was a dose-dependent increase in luciferase induction.

#### 2.3.3 Human Cell Line Activation Test (h-CLAT)

The h-CLAT is an *in vitro* skin sensitization assay addressing KE3 of the AOP for skin sensitization, the activation and mobilization of dendritic cells including induction of inflammatory cytokines and surface molecules leading to T-cell priming. The h-CLAT was performed at Burleson Research Technologies, Inc., according to OECD TG 442E (OECD, 2018b). The assay was conducted in the human monocytic leukemia cell line THP-1 and used flow cytometry to measure expression of CD86 (B7.2) and CD54 (intercellular adhesion molecule 1, ICAM-1) cell surface markers associated with dendritic cell activation. The positive control was 2,4-dinitrochlorobenzene prepared in DMSO and diluted to 4.0 μg/ml in culture medium. The negative control was culture medium with the appropriate solvent concentration added. Test substances were prepared based on solubility in either phosphate-buffered saline (PBS) or DMSO at final in-well concentrations up to 0.5% PBS or 0.1% DMSO. An eight-concentration dose range-finder cytotoxicity assay was conducted using propidium iodide staining to identify the concentration that resulted in 75% cell viability (25% cytotoxicity). If test substances prepared in PBS were not cytotoxic, the starting concentration was 0.5%. For the main assay, test substances were prepared in either PBS or DMSO at 100-fold (PBS) or 500-fold (DMSO) of 1.2 x the starting concentration producing 75% cell viability. Eight 1.2-fold dilutions in the appropriate solvents were made to obtain the stock solutions that were further diluted 50-fold (PBS) or 250-fold (DMSO) into the culture medium as working solutions, then diluted 2-fold in the plate to reach final in-well concentrations. CD86 and CD54 expression was measured by flow cytometry using fluorochrome-tagged antibodies. The relative fluorescence intensity for each marker, with respect to solvent controls, was determined at each of eight 1.2-fold dilutions of test material after a 24 h exposure to the test substance.

Each formulation was tested in at least two independent runs to derive a single result based on the CD86/CD54 expression levels. We considered a test substance to be positive if at least one of the following conditions were met in two independent runs:

• The relative fluorescence intensity for CD86 was greater than or equal to 150% in at least one tested concentration (with cell viability at least 50%).
• The relative fluorescence intensity for CD54 was greater than or equal to 200% in at least one tested concentration (with cell viability at least 50%).

For substances classified as positive, we determined the effective concentration that induced a relative fluorescence intensity of 150% for CD86 (EC150) and a relative fluorescence intensity of 200% for CD54 (EC200).

#### 2.3.4 *In Silico* Hazard Predictions

Read-across predictions for skin sensitization hazard were generated using OECD QSAR Toolbox v4.5, which is freely available software (OECD, 2021d). The simplified molecular-input line-entry system (SMILES) specifications of chemical structure and Chemical Abstracts Service registry numbers (CASRNs) for each formulation’s ingredients were used as inputs to QSAR Toolbox. Predictions for the ingredients were made using the automated workflow for “EC3 from LLNA or Skin sensitization from GPMT assays for defined approaches (SS AW for DASS).” If the automated workflow could not make a prediction because an ingredient was a salt, the salt was dissociated, and the automated workflow was applied to the organic portion of the ingredient to make a prediction; Toolbox does not make skin sensitization hazard predictions for inorganic structures. The Toolbox predictions for the formulation ingredients were then used to classify each formulation. Specifically, if a formulation contained one or more ingredients that were positive and the concentration of a positive ingredient in the formulation was at least 0.1%, then the formulation was classified as positive per the GHS guidance for determining the sensitization potential of mixtures (UN, 2021a). Otherwise, the prediction was negative. The Toolbox prediction for a formulation was considered inconclusive if it contained ingredients with no Toolbox prediction and ingredients with negative predictions only. Toolbox does not provide predictions for ingredients with undefined structures (e.g., substances of unknown or variable composition, complex reaction products, or biological materials).

### 2.4 Defined Approaches (DA) for Skin Sensitization

Data from the DPRA, KeratinoSens, h-CLAT, and Toolbox were used as information sources for multiple DAs. DAs utilize results from multiple non-animal information sources to achieve a predictive capacity for human skin sensitization potential that is equal to or greater than that of animal tests (OECD, 2021b). A DA consists of a fixed data interpretation procedure (e.g., mathematical model or rule-based approach) applied to specific *in silico*, *in vitro*, or *in chemico* data with adequate information to make a prediction on skin sensitization potential without expert judgment. An advantage of DAs for skin sensitization is that they utilize assays that address multiple KEs in the AOP. The limitations of DAs are based on the limitations of the individual data sources or information on a specific test substance.

#### 2.4.1 2 out of 3 DA (2o3)

The 2o3 DA is included in OECD Guideline 497 (OECD, 2021b), United States Environmental Protection Agency interim guidance (US EPA, 2018), and in European Chemicals Agency guidance (ECHA, 2021) for monoconsitutent substances, but is currently not accepted in the United Kingdom (UK HSE, 2021). It predicts skin sensitization hazard based on sequential testing, in no specific order, using the DPRA, KeratinoSens, and h-CLAT methods. The 2o3 DA requires concordant results from two assays to make a prediction. Thus, a test substance is classified as a sensitizer if the outcome in two assays is positive and negative if the outcome in two assays is negative. Borderline results, as defined in OECD Guideline 497 Annex 1, cannot be used as one of the two concordant tests.

We evaluated DPRA, KeratinoSens, and h-CLAT data to identify borderline results according to OECD Guideline 497 with small deviations. If one of the two concordant tests for the 2o3 was a borderline result, we considered the 2o3 prediction to be inconclusive. Because we did not have multiple independent DPRA runs for each substance, we used the single available run to evaluate whether it was a borderline result. For the KeratinoSens evaluation of borderline results, we used the average maximum fold induction of luciferase activity, EC1.5, and viability at the EC1.5. Statistical significance of the average maximum fold induction of luciferase activity compared with controls was not determined because the KeratinoSens data were generated before TG 442D was published and individual well data were no longer available. The evaluation of borderline results for h-CLAT adhered strictly to OECD Guideline 497 because these tests were recently performed, and all test data were available. Typically, negative h-CLAT results for substances with log Kow >3.5 cannot be used with confidence; however, log Kow is not applicable to mixtures such as the formulations we evaluated for this project.

#### 2.4.2 KE 3/1 Sequential Testing Strategy (STS)

The STS is accepted in the United States Environmental Protection Agency interim guidance (US EPA, 2018), but is not included in OECD Guideline 497 (OECD, 2021b) or agrochemical guidance from the European Chemicals Agency (ECHA, 2021) or the United Kingdom (UK HSE, 2021). It was originally described by Nukada et al. (2013) and addresses KEs 1 and 3 in the AOP for skin sensitization using the DPRA and h-CLAT, respectively. The STS provides both skin sensitization hazard and GHS potency classification, as illustrated in Table 1. A test substance is evaluated initially in the h-CLAT using the minimum induction threshold (MIT), which is the lowest value of the EC150 for CD86 induction or the EC200 for CD54 induction. A positive h-CLAT with MIT less than or equal to 10 μg/ml is predicted to be a GHS 1A sensitizer, while a substance having a MIT between 10 and 5,000 μg/ml is predicted to be a GHS 1B sensitizer. A negative h-CLAT result requires testing in the DPRA. A positive DPRA result predicts a GHS 1B sensitizer whereas a negative DPRA result yields a negative outcome for the DA with the test substance being considered GHS Not Classified (i.e., a nonsensitizer).

**TABLE 1:** The KE 3/1 sequential testing strategy (STS).

Testing Order	h-CLAT MIT (µg/mL)	DPRA	Hazard Classification	GHS Potency Classification
1^st^ h-CLAT	≤10	–	Positive	1A
	>10 to 5000	–	Positive	1B
	Negative	–	2^nd^ test in DPRA	–
2^nd^ DPRA	–	Positive	Positive	1B
	–	Negative	Negative	NC

DPRA, direct peptide reactivity assay; h-CLAT, human cell line activation test; MIT, minimum induction threshold.

#### 2.4.3 Integrated Testing Strategy v2 (ITSv2)

The ITSv2 is included in OECD Guideline 497 (OECD, 2021b) but not in United States Environmental Protection Agency interim guidance (US EPA, 2018) or in those for agrochemical formulations from the European Chemicals Agency (ECHA, 2021) or the United Kingdom (UK HSE, 2021). The ITSv2 addresses KE3 of the AOP using h-CLAT and KE1 using DPRA (OECD, 2021b). The ITSv2, which predicts both skin sensitization hazard and GHS potency classification, also incorporates an *in silico* hazard classification prediction from QSAR Toolbox. We selected ITSv2 for evaluation over ITSv1 because the *in silico* input is from freely available software. ITSv1 requires *in silico* input from a proprietary source, Derek Nexus v6.1.0.

The data interpretation procedure for the ITSv2 is based on a DPRA score calculated using the mean percent depletion of lysine and cysteine peptides or of the cysteine peptide only (in case of co-elution with the lysine peptide); an h-CLAT outcome based on the MIT; and a QSAR Toolbox skin sensitization prediction (Table 2). The scores for the individual inputs are summed and used to predict the skin sensitization hazard potential of a test substance (i.e., sensitizer vs. nonsensitizer) and the GHS potency classification (i.e., 1A, 1B, or Not Classified) (Table 3). The interpretation of the total score considers partial information situations in which one input is unavailable or out of domain. In some cases, potency category may not be assigned.

**TABLE 2:** ITSv2 scoring system for individual information sources.

Score	h-CLAT MIT (µg/ml)	DPRA mean cysteine and lysine depletion (%)	DPRA cysteine depletion (%)^a^	Toolbox prediction
3	≤10	≥42.47	≥98.24	-
2	>10 to ≤150	≥22.62 to <42.47	≥23.09 to <98.24	-
1	>150 to ≤5,000	≥6.38 to <22.62	≥13.89 to <23.09	Positive
0	Not calculated	<6.38	<13.89	Negative

a Cysteine-only depletion thresholds for DPRA are used in cases where (a) test substance co-elutes with the lysine peptide and (b) cysteine peptide depletion conforms to test acceptance criteria. There were no such cases in this study.

**TABLE 3:** Interpretation of total ITSv2 scores.

Total score	h-CLAT, DPRA, and Toolbox	h-CLAT and DPRA	h-CLAT or DPRA and Toolbox
6–7	1A	1A	-
5	1B	1^a^	-
3–4	1B	1B	1^a^
2	1B	1B	1B
1	Nonsensitizer	Inconclusive	Inconclusive
0	Nonsensitizer	Nonsensitizer	Inconclusive

a This score is positive and conclusive for hazard, but potency cannot be determined.

#### 2.4.4 Performance Analyses

The performance of the individual test methods and DAs for hazard classification was calculated by counting the number of true positive (TP), true negative (TN), false positive (FP), and false negative (FN) outcomes relative to the *in vivo* data. Accuracy, sensitivity, specificity, and balanced accuracy were calculated as follows:

$$\mathrm{Accuracy}\left(\%\right)=\left[\frac{\mathrm{TP+TN}}{\mathrm{TP+TN+FP+FN}}\right]\mathrm{*100}$$

Sensitivity(%)=[TP/(TP+FN)]*100

Specificity(%)=[TN/(TN+FP)]*100

Balanced Accuracy(%)=[Sensitivity(%)+Specificity(%)]/2

The performance of the STS or the ITSv2 DAs for GHS potency classification was determined by calculating the overall classification rate based on concordance with *in vivo* reference data and the concordant classification, underprediction, and overprediction rates for each GHS potency category (i.e., 1A, 1B, Not Classified). Inconclusive determinations were not included in performance calculations.

## 3 Results

### 3.1 Performance of the *In Vitro* and *In Silico* Methods for Hazard Classification

Supplementary File 3 provides the results from the individual methods–DPRA, KeratinoSens, h-CLAT, and QSAR Toolbox v4.5–as well as predictions from the 2o3, STS, and ITSv2 DAs for all tested agrochemical formulations relative to *in vivo* reference data for hazard and potency. These results were used to determine the performance of each test method or DA. No DPRA data were available for Dow6, due to test chemical interference with the peptide measurements, and for Dow8, which was not tested in the DPRA. The hazard predictions from QSAR Toolbox were inconclusive for six substances–Dow3, Dow7, Dow20, Dow23, Dow24 and Dow26–because the hazard predictions for the components were out of the domain of the read-across prediction or because a prediction could not be generated (Dow23).

Comparative performance data with respect to animal test results are shown in Table 4. Across the individual *in chemico* and *in vitro* methods, accuracy ranged from 52 to 81%, sensitivity from 45 to 92%, specificity from 20 to 87%, and balanced accuracy from 56 to 81%. KeratinoSens had the best overall performance, with accuracy of 81%, sensitivity of 75%, specificity of 87% and balanced accuracy of 81%. The QSAR Toolbox predictions alone had lower accuracy than the *in vitro* test methods (48%), with higher sensitivity (100%), but lower specificity (0%). However, there were no nonsensitizer hazard classifications for formulations based on read-across from QSAR Toolbox because any formulation ingredients that were classified as negative were either outside the applicability domain or combined with positive ingredients at greater than or equal to 0.1%.

**TABLE 4:** Performance of non-animal methods for GHS hazard classification in comparison with *in vivo* reference data.

Performance statistic	Individual methods				Defined approaches		
	DPRA (*n* = 25)	KeratinoSens (*n* = 27)	h-CLAT (*n* = 27)	QSAR Toolbox (*n* = 21)	2o3 (*n* = 19)	STS (*n* = 27)	ITSv2 (*n* = 24)
Accuracy (%)	64 (16/25)	81 (22/27)	52 (14/27)	48 (10/21)	79 (15/19)	52 (14/27)	54 (13/24)
Sensitivity (%)	45 (5/11)	75 (9/12)	92 (11/12)	100 (10/10)	90 (9/10)	92 (11/12)	91 (10/11)
Specificity (%)	79 (11/14)	87 (13/15)	20 (3/15)	0 (0/11)	67 (6/9)	20 (3/15)	23 (3/13)
Balanced Accuracy (%)	62	81	56	50	78	56	57

Borderline results were used in the assessment of the DPRA, KeratinoSens, and h-CLAT methods because the individual test guidelines do not recommend rejecting borderline results. The n for the 2o3 DA is reduced because borderline results were not used as one of the two concordant tests per OECD Guideline 497.

### 3.2 Performance of the Defined Approaches for Hazard Classification

As indicated in Section 2.4.1, borderline results for DPRA, KeratinoSens, and h-CLAT were not used for the 2o3 DA. There were three borderline results for DPRA, six borderline results for KeratinoSens, and nine borderline results for h-CLAT (Supplementary File 3). Five substances produced borderline results in more than one *in chemico/in vitro* method (Dow7, Dow10, Dow13, Dow16, and Dow22). The 2o3 DA had eight inconclusive results: Dow2, Dow6, Dow18, and the five formulations that had borderline results in more than one method. Dow2 and Dow18 had results that were negative for the DPRA, borderline in the KeratinoSens, and positive for the h-CLAT. Dow6 had no DPRA data and results that were positive for the h-CLAT, and negative for the KeratinoSens.

There were no inconclusive results for the STS DA and three inconclusive results for the ITSv2 DA. The inconclusive results for ITSv2 were for three substances, Dow7, Dow20, and Dow23, which had negative DPRA results and weakly positive h-CLAT results. Because the Toolbox predictions for these substances were inconclusive, the overall evaluation produced ITSv2 scores of 1, which were considered inconclusive based on OECD Guideline 497 (OECD, 2021b).

Across the DAs, accuracy ranged from 52 to 79%, sensitivity from 90 to 92%, specificity from 20 to 67% and balanced accuracy from 56 to 78%. The 2o3 DA had the best overall performance, with accuracy of 79%, sensitivity of 90%, specificity of 67%, and balanced accuracy of 78%. The predictive capacity of the 2o3 was similar to that of the KeratinoSens assay. While accuracy and balanced accuracy of the two approaches were similar (78–81%), the 2o3 had higher sensitivity (90 vs 75%) and lower specificity (67 vs 87%). The STS and ITSv2 results were driven by the h-CLAT outcomes and had similar performance statistics. The STS had exactly the same hazard outcomes for each substance as the h-CLAT, and the ITSv2 outcomes differed from h-CLAT outcomes only for formulations for which the ITSv2 DA produced inconclusive results.

### 3.3 Performance of the Defined Approaches for GHS Potency Classification

As previously noted, the GHS potency classifications for the *in vivo* reference data and STS and ITSv2 DAs for the agrochemical formulations are shown in Supplementary File 3. The 2o3 DA does not provide potency classifications as it only predicts hazard. Although Dow22 was positive for the CD54 marker in the h-CLAT, no EC200, and thus, no MIT, could be calculated due an inadequate dose-response curve. Because it was positive at the lowest dose tested, 1,200 μg/ml, we used this value as a surrogate MIT value. There were no inconclusive results for STS, and four substances had inconclusive results for ITSv2. Dow8 had positive h-CLAT and positive Toolbox results but no DPRA data. This resulted in a total score of 3, which is inconclusive for potency determination when DPRA data are missing. Three substances– Dow7, Dow20, and Dow23–had negative DPRA and positive h-CLAT data with inconclusive Toolbox results that yielded total ITSv2 scores of 1, and thus inconclusive ITSv2 potency results.

The correct overall classification rate, based on concurrence with *in vivo* reference data, for the two potency DAs was 52% for the STS and 43% for ITSv2 (Table 5). The overall underprediction rates were 4% for both DAs and the overall overprediction rates were 44% for STS and 52% for the ITSv2. Both DAs correctly classified the single GHS 1A sensitizer, thus there was no underprediction of this class. Neither DA overclassified nonsensitizer substances as GHS 1A sensitizers (Supplementary File 3). All misclassified substances were misclassified by one category. The STS was more successful at classifying GHS Category 1B sensitizers concordantly with *in vivo* tests than the ITSv2, with *in vivo* concordance for the two DAs being 91 and 67%, respectively. The STS misclassified one GHS Category 1B sensitizer while the ITSv2 misclassified three. Underprediction of GHS Category 1B sensitizers ranged from 9 to 11% and overprediction was 0% for the STS and 22% for ITSv2. Both DAs correctly classified nonsensitizers at 20–23%. However, nonsensitizers were overpredicted 77–80% by the DAs. Therefore, both DAs were likely to overclassify a test substance as GHS Category 1B that was GHS Not Classified based on *in vivo* data.

**TABLE 5:** Performance of the defined approaches for GHS potency classification in comparison with *in vivo* reference data.

Performance	STS				ITSv2			
	Overall (*n* = 27)	NC (*n* = 15)	1B (*n* = 11)	1A (*n* = 1)	Overall (*n* = 23)	NC (*n* = 13)	1B (*n* = 9)	1A (*n* = 1)
Correct Classification (%)	52 (14/27)	20 (3/15)	91 (10/11)	100 (1/1)	43 (10/23)	23 (3/13)	67 (6/9)	100 (1/1)
Underpredicted (%)	4 (1/27)	NA	9 (1/11)	0 (0/1)	4 (1/23)	NA	11 (1/9)	0 (0/1)
Overpredicted (%)	44 (12/27)	80 (12/15)	0 (0/11)	NA	52 (12/23)	77 (10/13)	22 (2/9)	NA

NC, GHS Not Classified (nonsensitizer); NA, not applicable.

### 3.4 Comparison Among Methods

As described, multiple non-animal (one *in chemico*, two *in vitro*, one *in silico* and three DAs) and animal-based (LLNA and guinea pig) test methods were used to assess the skin sensitization potential of 27 agrochemical formulations. Table 6 presents the results for all tested products based on each of the methods. In many cases, several non-animal approaches provided concordant results despite lack of agreement with the animal tests. While new approaches are typically evaluated with respect to the existing animal reference standard, there has been substantial evidence supporting the superior performance of methods and DAs that cover multiple KEs in the adverse outcome pathway for skin sensitization, as compared to human reference data (Kleinstreuer et al., 2018; OECD, 2021b). It is therefore appropriate that each of these methods, non-animal and animal alike, should be considered as potentially equivalent information sources when assessing skin sensitization hazard and potency predictions for a new data set.

**TABLE 6:** Skin sensitization results^a^ for 27 agrochemical formulations.

Code	DPRA hazard	h-CLAT hazard	KS hazard	QSAR TBv4.5 hazard	*In Vivo* hazard	2o3 hazard	ITSv2 hazard	STS hazard	*In Vivo* GHS potency	ITSv2 GHS potency	STS GHS potency
Dow1	0	BL 0	0	1	0	0	0	0	NC	NC	NC
Dow2	0	1	BL 0	1	0	INC	1	1	NC	1B	1B
Dow3	1	1	1	INC	1	1	1	1	1B	1B	1B
Dow4	1	BL 1	1	1	1	1	1	1	1B	1A	1B
Dow5	1	BL 1	1	1	1	1	1	1	1B	1A	1B
Dow6	INC	1	0	1	0	INC	1	1	NC	1B	1B
Dow7	BL 0	BL 1	0	INC	0	INC	INC	1	NC	INC	1B
Dow8	NT	1	1	1	1	1	1	1	1B	INC	1B
Dow9	1	1	0	1	1	1	1	1	1A	1A	1A
Dow10	BL 0	BL 0	0	1	0	INC	0	0	NC	NC	NC
Dow11	0	BL 1	0	1	0	0	1	1	NC	1B	1B
Dow12	0	BL 1	0	1	1	0	1	1	1B	1B	1B
Dow13	0	BL 1	BL 1	1	1	INC	1	1	1B	1B	1B
Dow14	0	1	0	1	0	0	1	1	NC	1B	1B
Dow15	1	1	1	1	1	1	1	1	1B	1B	1B
Dow16	0	BL 0	BL 0	1	1	INC	0	0	1B	NC	NC
Dow17	0	0	0	1	0	0	0	0	NC	NC	NC
Dow18	0	1	BL 0	1	0	INC	1	1	NC	1B	1B
Dow19	0	1	1	1	0	1	1	1	NC	1B	1B
Dow20	0	1	0	INC	0	0	INC	1	NC	INC	1B
Dow21	0	1	1	1	1	1	1	1	1B	1B	1B
Dow22	BL 1	1	BL 0	1	0	INC	1	1	NC	1B	1B
Dow23	0	1	1	INC	1	1	INC	1	1B	INC	1B
Dow24	1	1	BL 1	INC	0	1	1	1	NC	1B	1B
Dow25	0	1	0	1	0	0	1	1	NC	1B	1B
Dow26	1	1	0	INC	0	1	1	1	NC	1B	1B
Dow27	0	1	1	1	1	1	1	1	1B	1B	1B

a Results from three in chemico/*in vitro* assays (yellow), one in silico model (blue), historical animal reference data (green), and three DAs (orange) providing both hazard and potency predictions. 0, negative; 1, positive, BL, borderline; INC, inconclusive; KS, KeratinoSens; NC, GHS Not Classified (nonsensitizer); NT, not tested; TB, Toolbox.

## 4 Discussion

One current limitation to the regulatory application of DAs is that the non-animal methods that comprise them have only been evaluated using monoconstituent substances rather than mixtures or product formulations. With this study, we sought to expand the applicability of internationally accepted OECD test methods and DAs by generating skin sensitization hazard and potency assessments for 27 water-based or solvent-based agrochemical formulations. We evaluated three rule-based DAs: the 2o3, the KE 3/1 STS, and the ITSv2. The 2o3 and the ITSv2 have been adopted for hazard classification and GHS potency classification by OECD (OECD, 2021b), and the 2o3 and the STS are accepted for hazard classification by the United States Environmental Protection Agency (US EPA, 2018), although the STS also classifies substances in GHS potency categories (Nukada et al., 2013). The DAs combine skin sensitization potential information from three non-animal methods that map to key events of the skin sensitization adverse outcome pathway–the DPRA, the KeratinoSens assay, and the h-CLAT–as well as *in silico* hazard predictions from QSAR Toolbox v4.5. There is limited information on the applicability of the individual methods to agrochemical formulations aside from a small proof of concept for KeratinoSens (Settivari et al., 2015).

We evaluated the individual non-animal methods as stand-alone methods for hazard classification of the agrochemical products to compare their performances with the DAs, all with respect to historical reference animal test data. Of the individual methods, KeratinoSens performed best in predicting *in vivo* hazard outcomes (Table 4). The best performing DA was the 2o3. However, the 2o3 DA did not outperform the KeratinoSens assay alone with respect to accuracy or balanced accuracy. Both KeratinoSens and the 2o3 DA had accuracy and balanced accuracy of 78–81%. The 2o3 DA had higher sensitivity (90%) than specificity (67%) whereas the KeratinoSens had a better balance of sensitivity (75%) and specificity (87%). The balanced accuracy of the 2o3 DA in this study, 78%, was less than that reported in OECD Guideline 497 for 134 monoconstituent substances (84%), which yielded sensitivity of 82%, and specificity of 85% as compared to LLNA reference data (OECD, 2021b). Relative to animal data, the 2o3 DA had higher sensitivity than specificity for classification of the agrochemical formulations.

For our data set of agrochemicals, the STS and the ITSv2 produced very similar results for *in vivo* hazard classification because both DAs rely on DPRA and h-CLAT results (Table 4). These DAs had balanced accuracies of 56–57%, which were lower than that for the 2o3 DA. Sensitivities were 91–92% and specificities were 20–23%. Thus, these DAs were much better at classifying sensitizers than nonsensitizers. The STS and ITSv2 results were driven by the h-CLAT and had similar performance statistics. The STS had exactly the same hazard outcomes for each substance as the h-CLAT and the ITSv2 DA differed with h-CLAT only for formulations for which the ITSv2 had inconclusive results (Supplementary File 3). Concordance of the ITSv2 classification with reference animal data was higher for the 156 monoconstituent substances reported in OECD Guideline 497, than that of the current set of agrochemical formulations, especially with respect to balanced accuracy (80%) and specificity (67%), but sensitivity was similar (93%) (OECD, 2021b). It is not unusual for the h-CLAT to perform with higher sensitivity than specificity or accuracy in other datasets (Urbisch et al., 2015; Hoffmann et al., 2018; OECD, 2021b). We speculate that some overprediction could be due to the presence of substances of natural origin, such as lipopolysaccharide, in the formulations. The h-CLAT may be overly sensitive to the stimulation of the CD86 and CD54 cell markers by endotoxins/liposaccharides (Tsukumo et al., 2018; Kobayashi-Tsukumo et al., 2019). It is not uncommon for agrochemical formulations to have active ingredients that are fermentation products or co-formulants/inerts of natural origin (such as methylated seed oil used as surfactant enhancing foliar absorption). This should be a point of caution when using h-CLAT.

Application of the borderline ranges for the 2o3 as described in OECD Guideline 497 improved its performance with respect to the animal data but yielded eight inconclusive results. Before excluding borderline results, the balanced accuracy of the 2o3 was 73%, sensitivity was 75% and specificity was 71% (data not shown). After excluding borderline results, balanced accuracy increased to 78%, sensitivity increased to 90% and specificity decreased slightly, to 67%. The exclusion of borderline values in the OECD evaluation of the 2o3 using single constituent substances had a similar effect (OECD, 2021e). It reduced the reference data set (from 168 to 134 chemicals) and increased balanced accuracy from 79 to 84%, increased sensitivity from 74 to 82%, and left specificity unchanged at 85%, in comparison with LLNA data (OECD, 2021e).

For GHS potency categorization, the performance of the individual test methods could not be compared with that of the STS and ITSv2 because the individual methods have not been validated for potency determination. The performances of the STS and ITSv2 were very similar for predicting *in vivo* GHS potency categories (Table 5). Both DAs derive potency information from the h-CLAT. Both also use DPRA, but only the ITSv2 uses DPRA for potency information, although use of these data did not improve the performance of the ITSv2 over the STS for this set of agrochemicals. The overall correct classification rates of the STS and ITSv2 DAs were 52 and 43%, respectively. The overall underprediction rates (4%) were much lower than overprediction rates (44–52%). The overprediction of *in vivo* nonsensitizers as GHS 1B sensitizers contributed greatly to the underprediction rate. The overprediction rate of nonsensitizers was 80% for the STS and 77% for the ITSv2. A previous evaluation of the STS for potency classification of monoconstituent substances relative to LLNA data reported a higher correct classification rate (71%) and more balanced over- and underprediction rates with 12% overprediction and 18% underprediction (Takenouchi et al., 2015). The performance of the ITSv2 against LLNA data reported in OECD Guideline 497 was also better for the 141 monoconstituent substances evaluated by the OECD; correct classification rates were 72% for GHS 1A and 1B sensitizers and 67% for nonsensitizers (OECD, 2021b). For the agrochemicals, the correct classification rates were 100% for GHS 1A (which had only one substance), 67% for GHS 1B sensitizers, and 23% for nonsensitizers.

We recognize that the current study has several limitations in its evaluation of the performance of DAs for predicting human skin sensitization potential and potency of agrochemical products. One limitation is the small number of substances evaluated. Another is that all 27 substances were either water- or solvent-based formulations, so we do not know how relevant these results are to other agrochemical formulation types. A third is that our reference data consisted of animal data rather than human data. There is evidence that the animal studies may not accurately predict human endpoints (Kleinstreuer et al., 2018). The OECD evaluation of monoconstituent substances showed that the performance of the LLNA for predicting human skin sensitization hazard yielded 58% for balanced accuracy, 94% for sensitivity, and 22% for specificity (OECD, 2021b). The absence of human data for the agrochemical formulations beyond case studies or adverse event information with questionable reliability presents a challenge to assessing the true performance of the DAs to predict human skin sensitization potential. Although we used the best reference data available, the agreement of the animal data with human responses is uncertain.

A further limitation of our study was the retrospective application of DAs to the data, which resulted in more inconclusive predictions than would be obtained using a prospective approach. Inconclusive predictions for the 2o3 DA were obtained for 30% (8/27) of the formulations due to borderline results of the individual test methods. In a prospective testing situation, an investigator would be able to perform additional tests as borderline results were produced to minimize the number of final discordant results. For example, if two KeratinoSens runs had been conducted, but one produced a borderline result, OECD Guideline 497 would require a third repetition. If the third repetition was not a borderline result, a final positive or negative call could be made, rather than an inconclusive result. Despite this limitation, inconclusive DA predictions may be considered in a weight-of-evidence approach or within IATA to reach a hazard classification decision or develop a risk assessment. Other information considered might include demonstration of exposure to the test system, existing *in vivo* data, clinical data, read-across, and other *in vitro/in chemico/in silico* data (OECD, 2021b).

Our evaluation shows that the 2o3 DA has the most promising performance for predicting the animal-based hazard classification of these particular agrochemicals. KeratinoSens had slightly higher balanced accuracy compared with the 2o3 DA (81 vs. 78%), however, the sensitivity was lower than that of the 2o3 DA (75 vs. 90%) and the specificity was higher (87 vs. 67%). We have more confidence in the results of 2o3 DA because it assesses at least two key events of the AOP. The performance of the 2o3 DA was more similar to its performance in the classification of monoconstituent substances (OECD, 2021b) than the performance of the STS or the ITSv2 (Takenouchi et al., 2015; OECD, 2021b). Given the theoretical advantages of DAs over individual *in vitro* methods and their previously reported success in classifying monoconstituent substances, the low concordance with reference *in vivo* GHS potency classifications for this set of agrochemical formulations was somewhat surprising. However, given the biological and mechanistic relevance of the DAs and their demonstrated superior performance when compared to available human reference data, it cannot be ruled out that the historical animal results may actually be incorrect, and the DAs may provide a more human health protective outcome. Further investigation, including testing of more and additional types of agrochemicals, will be required to determine whether our results with these DAs are applicable to other agrochemical formulations or other mixtures.

**FIGURE 1:**
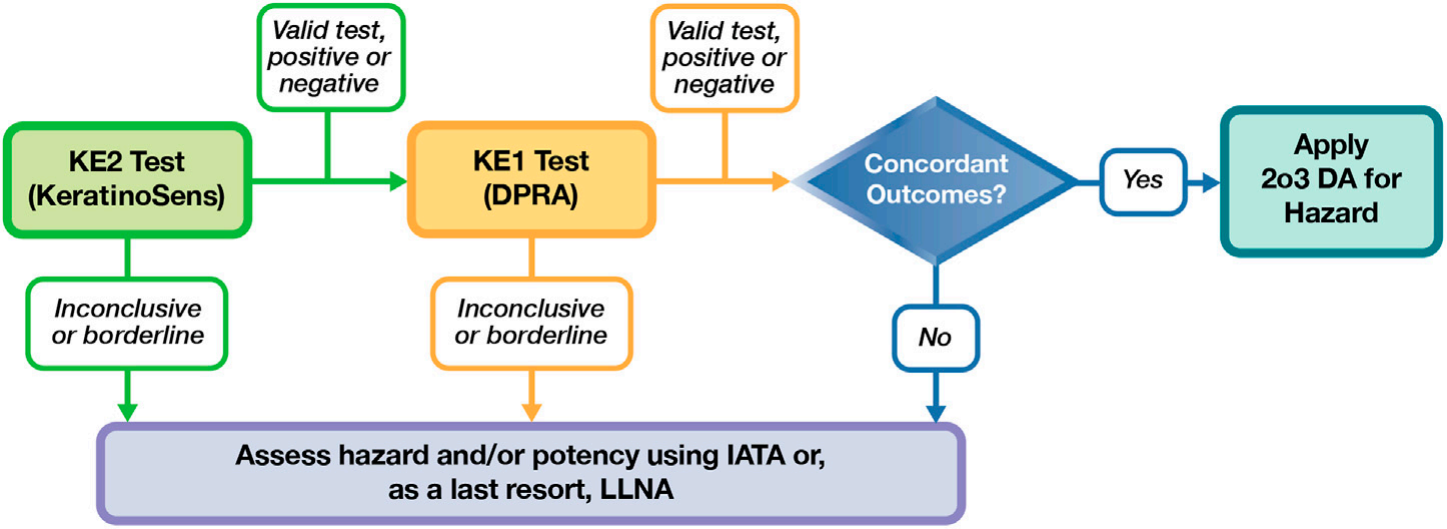
Proposed framework for a non-animal assessment of skin sensitization potential of agrochemical formulations.

In conclusion, based on the limited amount of information available today in the literature and in this study, we identified a potential prospective testing strategy shown in Figure 1. This could be considered a first “work in progress” step using the existing methods that demonstrated higher applicability and reliability in this study, KeratinoSens and DPRA. If these two tests are applicable and the results are concordant, the 2o3 approach could be used and additional *in vitro* or *in vivo* testing would not be necessary. In our database, only 11/27 substances had concordant results for the KeratinoSens and DPRA tests. While the number of applicable substances was limited, the performance of the 2o3 was good. Sensitivity was 100% (4/4), specificity was 86% (6/7), balanced accuracy was 93%, and accuracy was 91%. Substances where the KE2 and KE1 test results are not applicable and concordant would undergo additional testing and/or an assessment under IATA. This initial exercise highlights possible routes to reduce animal use and identifies further research needs to characterize test methods and DAs applicable for agrochemical formulations. These may include, for example, KE3 assays that have balanced predictivity, KE1 assays with higher compatibility with agrochemical formulations, data sharing exercises with existing paired *in vitro-in vivo* testing, more complex models (e.g., 3D skin, genomic signatures), and additional testing on multiple formulation types to establish broader applicability.

## Data Availability

The original contributions presented in the study are included in the article/Supplementary Material, further inquiries can be directed to the corresponding author.

## Abbreviations

2o3, 2 out of 3; AOP, adverse outcome pathway; DA, defined approach; DMSO, dimethyl sulfoxide; DPRA, direct peptide reactivity assay; EC1.5, concentration of test substance increasing luciferase activity by 1.5 fold; EC150, concentration of a test substance inducing a relative fluorescence intensity of 150% for CD86; EC200, concentration of a test substance inducing a relative fluorescence intensity of 200% for CD54; FN, false negative; FP, false positive; GHS, United Nations Globally Harmonized System of Classification and Labelling of Chemicals; h-CLAT, human cell line activation test; IATA, integrated approaches to testing and assessment; ITS, integrated testing strategy; KE, key event; LLNA, murine local lymph node assay; MIT, minimum induction threshold; OECD, Organisation for Economic Co-operation and Development; PBS, phosphate-buffered saline; STS, sequential testing strategy; TN, true negative; TP, true positive.
